# Nectary Structure and Nectar Secretion Characteristics Among Various Cultivars of *Paeonia lactiflora*

**DOI:** 10.3390/plants15040580

**Published:** 2026-02-12

**Authors:** Hui Cai, Wenjie Ma, Yingling Wan, Yan Liu

**Affiliations:** Beijing Key Laboratory of Ornamental Plants Germplasm Innovation & Molecular Breeding, Beijing Laboratory of Urban and Rural Ecological Environment, National Engineering Research Center for Floriculture, School of Landscape Architecture, Beijing Forestry University, Beijing 100083, China; cai_hui111@163.com (H.C.); wan_yingling@bjfu.edu.cn (Y.W.)

**Keywords:** anatomical structure, stomata, vascular bundle, sugar components, photosynthetic potential

## Abstract

Background: *Paeonia lactiflora* Pall. produces substantial quantities of nectar during the bud stage. In the production of cut flowers, this nectar attracts contaminants that compromise the quality of the flowers. The current practice of rinsing flowers with clean water escalates production costs. Consequently, reducing nectar secretion during the bud stage has emerged as a significant technical challenge for the industry. Nonetheless, insufficient fundamental knowledge concerning the structure of *P. lactiflora* nectaries and the physiology of nectar secretion impedes the development of pertinent regulatory technologies. Methods: This study established a “nectar secretion index” to evaluate nectar production in various *P. lactiflora* cultivars. Nectar sugar concentration and composition were measured using a refractometer and gas chromatography–mass spectrometry (GC-MS). Observations of changes in nectary epidermal morphology and anatomical structure during nectar secretion were conducted using scanning electron microscopy and light microscopy. Key Results: The quantity of nectar secreted by various *P. lactiflora* cultivars can differ. The indices were not significantly correlated with flowering period, flower color, or flower type. At the peak of nectar secretion, the sugar concentration of nectar secretion by different cultivars’ flower buds varied. Sucrose is the primary sugar component in this nectar. Nectar is secreted along the basal margins of the bracts and sepals on the abaxial surface of all cultivars. Specialized raised stomata are located on the upper epidermis, through which nectar is secreted. In contrast, the epidermal stomata located outside nectar-secreting areas exhibit a normal morphology. Specialized stomata do not secrete nectar concurrently. The stomatal aperture and the percentage of nectar-secreting stomata at the secretion sites are significantly higher in high-nectar-producing cultivars than in low-nectar-producing cultivars. Anatomical observations of bract nectaries indicate that, irrespective of nectar production levels, specialized stomata are consistently located adjacent to vascular bundles. During the initial stage of nectar secretion, no starch was detected in the bract nectaries. In contrast, the stomata in non-secretory epidermal cells of bracts maintain a normal morphology, and calcium oxalate crystals were observed within the subepidermal tissues. Throughout the nectar secretion process, the content of photosynthetic pigments and the Fv/Fm ratio in the bracts and sepals of various cultivars correlated with nectar secretion volume. Conclusions: This study, informed by observations of numerous *P. lactiflora* cultivars, elucidates the structural characteristics of its nectaries and the nectar secretion properties of various cultivars during the bud stage. It confirms that these nectaries are classified as extrafloral nectaries, specifically structural nectaries consisting of specialized raised stomata and closely associated vascular bundles beneath them. No significant differences in nectary structure or location were noted among cultivars with differing nectar yields. However, both the aperture of nectary stomata and the percentage of nectar-secreting stomata exhibited a significant positive correlation with secretion levels. The intrinsic photosynthetic potential at the nectary sites varies significantly among cultivars. The nectar is not derived from stored cellular starch but likely originates simultaneously from both photosynthesis and phloem transport. These findings provide a theoretical foundation for the development of subsequent regulatory technologies.

## 1. Introduction

*Paeonia lactiflora* Pall. is a perennial herb belonging to the Paeoniaceae family, which encompasses the genus *Paeonia*. In recent years, the market demand for this important cut flower has increased annually [1]. Research has shown that its buds can secrete nectar during the developmental stage, while nectar secretion stops during flowering. This phenomenon suggests that its nectar secretion behavior may serve a defensive function [2,3]. However, the copious nectar secretion from the flower buds captures airborne dust and small insects on the buds or upper leaf surfaces. This increases susceptibility to sooty and gray mold diseases, thereby heightening the risk of losses during storage and transportation. Post-harvest washing alone is insufficient to completely eliminate these impurities from the buds. Improper shade-drying further compromises subsequent flowering, significantly degrading the quality of *P. lactiflora* cut flowers [4]. However, research on its nectaries and nectar secretion remains limited, and there is a lack of foundational data to effectively support the development of nectar regulation technologies.

Nectaries are specialized tissues that are attached to the surface of a plant’s nutritional or reproductive organs, responsible for nectar secretion and constituting part of these organs [5]. Currently, nectaries are not solely classified based on their location as floral (FNs) or extrafloral nectaries (EFNs). Some nectaries situated on floral organs that do not participate in pollination are also categorized as EFNs [6,7,8,9,10]. Based on their structure, nectaries can be classified into two types: structural and non-structural. Structural nectaries consist of a secretory epidermis, nectariferous tissue, and vascular bundles, whereas non-structural nectaries contain only a secretory epidermis [11]. Nectaries can also be categorized based on their starch content as starch-storage nectaries or non-starch-storage nectaries. The former accumulate starch prior to nectar secretion, with the degraded starch serving as the nectar source [12], while the latter show no starch accumulation before secretion and derive pre-nectar directly from immediate photosynthesis [13]. After accumulating in nectaries, nectar is released through various mechanisms. Most plants secrete nectar through modified stomata [14,15]. Other methods include secretion through specialized trichomes [16] or cuticle rupture [17]. Some plants utilize two or more secretion pathways [18,19].

To date, literature reports on *P. lactiflora* nectar secretion remain scarce. One study utilizing ‘Hang bai shao’ as the research material revealed that the volume of nectar secreted shows a positive correlation with leaf count and plant height. Plants cultivated in full sunlight exhibited higher nectar secretion compared to those grown in shaded environments. *Paeonia lactiflora* nectaries have been classified as either extrafloral (leaf) or floral (bract or sepal), indicating that nectar secretion may represent an adaptation to environmental conditions or an expression of nutrient surplus [20]. Another study on ‘Da Fu Gui’ found that nectar was secreted from bud emergence to flowering, with the lowest secretion observed during blooming. This indicates that its nectar production may function as a defense mechanism against herbivores. This study categorized nectaries as either extrafloral (bracts) or floral (sepals) [21]. However, the two researchers arrived at different conclusions regarding the classification of these nectaries and the biological significance of nectar secretion, despite both basing their observations on a single cultivar. Although Dong-Xu Huang [22] conducted quantitative analyses of nectar secretion across multiple *P. lactiflora* cultivars, the observation period was set at the color-showing stage (i.e., the late phase of nectar secretion). Consequently, substantial nectar secretion from the bracts during the early stages could not be recorded. Overall, existing studies not only exhibit discrepancies in the classification criteria for nectary types in *P. lactiflora* but also offer divergent interpretations regarding the physiological significance of nectar secretion. Furthermore, the precise morphological and anatomical structure of the nectaries during development remains unelucidated, while systematic comparative studies on nectar secretion characteristics across different cultivars remain notably scarce. Therefore, further exploration of the anatomical structure and secretion mechanisms of these nectaries is necessary to provide the theoretical foundations for developing relevant regulatory technologies and ultimately facilitating the effective management of *P. lactiflora* nectar production.

The objectives of this study are threefold: (1) to investigate the patterns of nectar secretion in *P. lactiflora* by observing the timing, location, and quantity of nectar secretion across 32 cultivars; (2) to compare structural differences in nectary development among cultivars with varying nectar yields; (3) to examine the effects of nectary photosynthetic potential and the application of exogenous hormones on nectar secretion in its flowers during secretion. This study aims to inform the development of technologies to regulate nectar secretion in *P. lactiflora*.

## 2. Material and Methods

### 2.1. Plant Material

The 32 cultivars of *P. lactiflora* used in the test are shown in Table 1. The tested cultivars were planted in the open fields of Mudan District, Heze City, Shandong Province, and the plants were 3–4-year-old divided seedings.

### 2.2. Stages of Nectar Secretion

**Figure 1:**
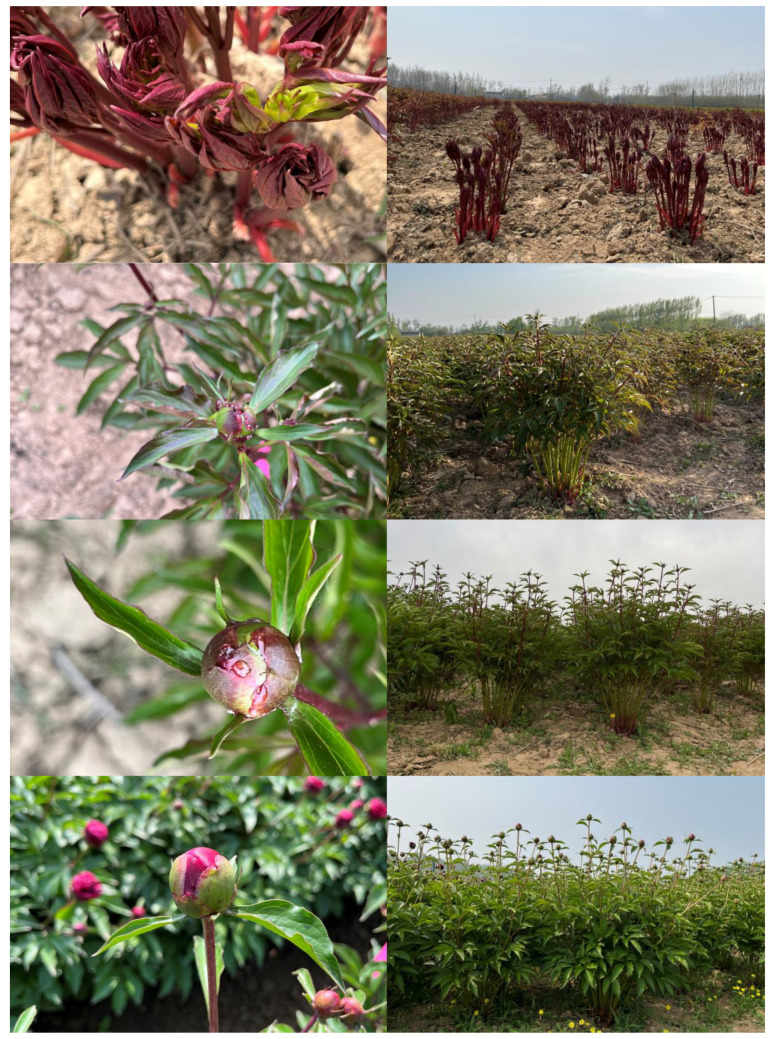

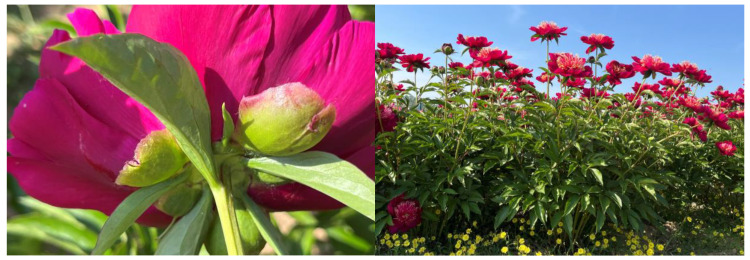
Correspondence between the nectar secretion stages and the growth phases of JXSS. Note: The period of nectar secretion is denoted by S1–S5, while the *P. lactiflora* growth phase is represented by P1, P3, P4, P7, and P10.

Drawing on Li-Jun Niu’s [4] classification of *P. lactiflora* cultivation requirements for cut flowers and the criteria established by Fang-Yun Cheng [26] for defining its bud maturity and flowering stages, we have developed a unified framework that integrates both. The early stem growth phase (P1) is identified as stage S1 of nectar secretion. During this stage, flower buds are almost completely enclosed by bracts, and no nectar is secreted. The stem and leaf growth stage (P3) commences when the first outer sepal is fully exposed and the second sepal is slightly visible. The flowers of *P. lactiflora* then begin to secrete nectar, which is designated as stage S2. The pre-hard-bud stage (P4) occurs when both sepals are fully exposed, accompanied by significant nectar secretion and visible dripping nectar. This stage is designated as S3. The color change stage (P7) involves the exposure of inner petals displaying vivid coloration and a firm bud body with a slightly softened apex. This marks the late nectar secretion phase (S4). Full bloom (P10) is designated as the final nectar secretion stage (S5). Taking the cultivar ‘Jin Xing Shan Shuo’ as an example, Figure 1 demonstrates the growth stages that correspond to its nectar secretion phases.

### 2.3. Measurement of Daily Nectar Production

Nectar in *P. lactiflora* forms spherical droplets along the margins of bracts and sepals. This study quantified nectar production by recording both the number and size of droplets produced by various cultivars [27]. Daily nectar production was measured by removing the previous day’s residual nectar using clean water at 08:00 each morning and quantifying the volume of nectar present at 08:00 the following day. For each cultivar, ten randomly selected plants were sampled from three separate experimental plots. Three flowers were randomly selected from each plant, resulting in a total of 90 flowers examined per cultivar. The results are expressed as the mean ± standard deviation [28]. Buds measured at each stage were excluded to mitigate the influence of ‘bud washing’ on nectar secretion.

### 2.4. ‘Nectar Secretion Index’ for the Rapid Assessment of P. lactiflora Nectar Production

**Figure 2:**
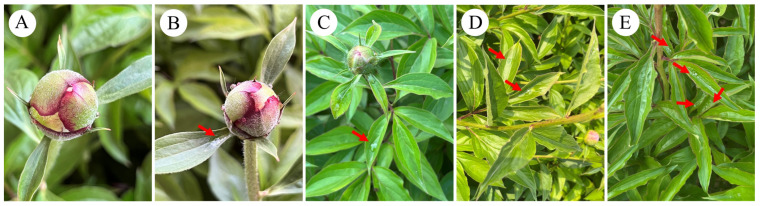
Classification of nectar secretion in *P. lactiflora*. Note: (**A**) Grade I, little nectar secretion, no nectar drops; (**B**) Grade II, bract leaf or single leaf nectar storage, no nectar drops; (**C**) Grade III, compound leaves with 1 drop of nectar; (**D**) Grade IV, compound leaves with 2~3 drops of nectar; (**E**) Grade V, compound leaves with more than 3 drops of nectar.

Nectar secretion level was classified based on the nectar dripping observed in various cultivars at the peak nectar secretion stage (S3) (Figure 2). The following classification was used: Grade I, little nectar, no dripping phenomenon (Figure 2A); Grade II, bracts or single leaves stored nectar, no dripping phenomenon (Figure 2B); Grade III, 1 drop of nectar on compound leaves (Figure 2C); Grade IV, 2 to 3 drops of nectar on compound leaves (Figure 2D); Grade V, more than 3 drops of nectar on compound leaves (Figure 2E). Fifteen plants were selected from each cultivar, and 3 flowers were randomly chosen from each plant to record the nectar dripping situation. This observation was repeated 3 times, with a total of 45 plants and 90 flowers. The ‘nectar secretion index’ was calculated following Xu et al. [29], and the formula was as follows:

$$\mathrm{Nectar}\;\mathrm{secretion}\;\mathrm{index}=\sum \left(xn\right)/(AN)\times 100\%$$

where “*x*” denotes the nectar secretion level, “*n*” indicates the corresponding number of flowering branches, “*A*” represents the maximum nectar secretion level, and “*N*” signifies the total number of examined flowering branches. The ‘nectar secretion index’ reflects the plant’s nectar production; a higher index indicates greater nectar production.

### 2.5. Determination of Nectar Sugar Concentration

At the peak of nectar secretion from flower buds (S3) in 2022 and 2023, 60 flower buds were randomly selected from each cultivar, which was repeated 6 times. The nectar from a total of 360 flower buds was collected. Before nectar collection, each flower bud used in the experiments was carefully cleaned with deionized water, and the nectar secreted on the day of collection was gathered. The collected nectar was stored in 1.5 mL centrifuge tubes. Following collection, an appropriate amount of nectar was dropped onto the prismatic surface of a handheld refractometer (0–95% Brix; Nohawk, Shenzhen, China) to measure the concentration of soluble solids [6].

### 2.6. Analysis of P. lactiflora Nectar Sugar Components by GC-MS

Gas chromatography–tandem mass spectrometry (GC-MS) was employed to conduct a precise qualitative and quantitative analysis to identify 32 sugar components in the nectar. These components included monosaccharides, such as glucose, D-fructose, and inositol; disaccharides, such as sucrose, maltose, and trehalose; and trisaccharides, such as raffinose. The GC-MS instrument utilized in this study was an Agilent 8890-5977B (Agilent Technologies, Santa Clara, CA, USA). The DB-5MS column (Agilent Technologies, Santa Clara, CA, USA, 30 m × 0.25 mm × 0.25 μm) employed helium as the carrier gas at a flow rate of 1 mL/min, with a programmed temperature ramp of 160–200–270–300–320 °C and an injection volume of 1 μL. Three replicates were conducted for each cultivar [30,31].

### 2.7. Ultrastructural Observation

Scanning electron microscopy (SEM) was employed to examine the surface morphology of the bracts from S1–S5 and sepals at developmental stage S3 of *P. lactiflora* cultivars with different nectar yields. The specimens were fixed overnight in 2.5% glutaraldehyde in 0.1 M phosphate buffer at pH 7.2–7.4 at 4 °C, dehydrated through a series of alcohols, critical-point-dried, gold-coated, and then examined and photographed using an SU-8010 cold field emission scanning electron microscope (Hitachi, Tokyo, Japan). Stomatal length (SL), stomatal width (SW), stomatal area (SA’), and stomatal aperture (SA), defined as the width of the widest part of the inner wall of the stomata, were measured via image analysis (Supplementary Figure S1) [32]. The number of stomata within the field of view was recorded, and the stomatal density (SD) was calculated as the number of stomata per unit area of the field of view. Additionally, the number of stomata exhibiting nectar secretion within the field of view was recorded, and their percentage was determined: percentage of nectar-secreting stomata (SS) = (number of nectar-secreting stomata/total number of stomata) × 100% [33].

### 2.8. Anatomical Structure Observation

The nectary margins and non-marginal tissues of the bracts were excised using a sterile blade and immediately fixed in an FAA fixative solution (formaldehyde:glacial acetic acid:70% ethanol = 1:1:18, *v*/*v*/*v*) for a minimum of 24 h. Subsequently, dehydration and embedding were performed in accordance with standard paraffin sectioning protocols [34]. Sections (6 μm) were then cut on a LEICA RM 2235 (Leica Microsystems, Germany, Wetzlar) rotary microtome for subsequent staining and observation [35]. The tissues were stained using two methods: (1) 0.05% toluidine blue to observe the anatomical structure [36] and (2) periodate/Schiff’s reagent to assess the dynamics of starch [37]. The samples were observed and photographed using a LEICA DM2500 microscope (Leica Microsystems, Germany, Wetzlar).

### 2.9. Determination of Photosynthetic Pigments and Assessment of Photosynthetic Potential

An acetone–ethanol mixture (1:1, *v*/*v*) was used to extract photosynthetic pigments from *P. lactiflora* tissues (single leaves and the 3rd to 5th south-facing apical compound leaves, bracts, and sepals). The samples were kept in the solution in darkness and left until the tissues became completely white, typically after 24 h. Measurements were conducted at 663 nm, 646 nm, and 470 nm using a UV spectrophotometer (BioMate 3S, Thermo Fisher Scientific, Waltham, MA, USA). The chlorophyll fluorescence parameters (Fv/Fm) of the leaves (specifically, the third to fifth south-facing compound leaves at the apex), bracts, and sepals at the S3 stage were measured using a chlorophyll fluorometer (PAM-2500, Walz, Effeltrich, Germany). Specifically, the plant tissue was spread flat in a fluorescence clamp to cover the 4 mm^2^ test aperture. After 30 min of dark adaptation, the analysis probe was placed on the leaf clamp, and measurements were taken with the light shield open. In this context, Fv represents the variable, Fm denotes the maximum fluorescence of dark-adapted samples, and Fv/Fm indicates the maximum quantum efficiency of the sample under 0 PPFD conditions.

### 2.10. Flower Bud Spraying Treatment

During the S2 stage, two cultivars (‘Lan Hai Bi Bo’ and ‘Yang Fei Chu Yu’) were treated with solutions of 0.5 mmol/L methyl jasmonate (MeJA) and 2 mmol/L phenidone [38,39,40]. All working solutions (including the ethanol/Tween-20 control) were formulated in anhydrous ethanol containing 0.02% (*v*/*v*) Tween-20 and diluted to volume with distilled water. Each treatment consisted of 45 plants, with three biological replicates of 15 plants each. The solutions were uniformly sprayed onto the surfaces of the flower buds using a pressure sprayer until a continuous liquid stream was formed. Spraying was repeated after 30 min to ensure adequate absorption [41]. Nectar secretion from *P. lactiflora* flower buds was quantified at the S3 stage, which occurred 7 days post-treatment.

### 2.11. Statistical Analysis

Experimental data were compiled using Excel 2019, and statistical analyses were conducted with SPSS 26.0. Data were expressed as means ± standard deviations. An independent-samples t-test was employed to determine the significance of differences between the two groups, while one-way ANOVA was used for comparisons involving three or more groups. Non-parametric tests were applied when the data did not meet ANOVA assumptions. Significance was defined as *p* < 0.05, and correlations were assessed using Spearman correlation analysis. Data visualization was performed using Origin 2021.

## 3. Results

### 3.1. The Duration of Nectar Secretion Across Different P. lactiflora Cultivars

**Figure 3:**
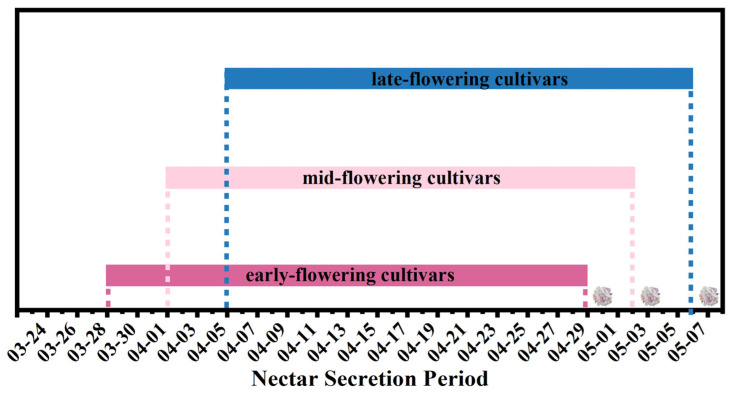
Nectar secretion periods of *P. lactiflora* cultivars with different flowering times. Note: The flowers are arranged in chronological order (from left to right) to illustrate the full-bloom stages of early-, mid-, and late-flowering cultivars.

Observations indicate that *P. lactiflora* in Heze City, Shandong Province, naturally blooms from mid-to-late April to early May. All cultivars secrete nectar prior to blooming, with this period ranging from late March to early May. Each cultivar exhibits a nectar secretion period of up to one month. The only difference between *P. lactiflora* with varying flowering times is their nectar secretion period: early-flowering cultivars secrete nectar from 28 March to 29 April, mid-flowering cultivars from 1 April to 2 May, and late-flowering cultivars from 5 April to 6 May (Figure 3).

**Figure 4:**
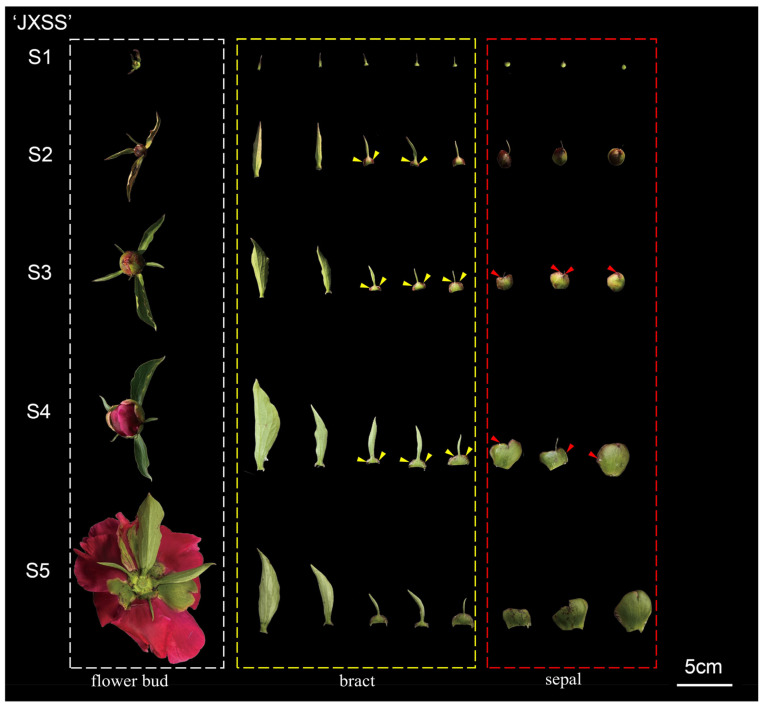
The nectariferous period of JXSS. Note: The yellow triangle denotes nectar on the bract, and the red triangle denotes nectar on the sepal. The white, yellow, and red boxes represent the flower bud, bract, and sepal, respectively.

There are no differences in the nectar secretion sites and processes among different cultivars. Figure 4 illustrates this for the cultivar ‘Jin Xing Shan Shuo’. As the flower bud develops, nectar initially appears along the apical edge of the swollen base of the bract. Once the sepals are no longer covered by the bract, nectar is also secreted along the apical edge of the sepals. Based on nectar secretion levels, the nectar secretion process can be categorized into five stages (S1–S5), demonstrating an overall trend of increasing followed by decreasing secretion. Taking the cultivar ‘Jin Xing Shan Shuo’ as an example (Figure 4): S1: Pre-Secretion Stage: No visible nectar is observed. S2: Initial Nectar Secretion Stage: Visible nectar droplets appear along the edges of the swollen basal regions of the bracts. S3: Peak Nectar Secretion Stage: Simultaneous nectar secretion occurs along the edges of both bracts and sepals. Cultivars with high secretion exhibit varying degrees of dripping. S4: Late Nectar Secretion Stage: The volume of secretion decreases, yet secretion persists along the margins of the bracts and sepals. S5: Terminal Nectar Secretion Stage: Nectar secretion ceases.

### 3.2. Nectar Production Varied Significantly Among Different P. lactiflora Cultivars

**Figure 5:**
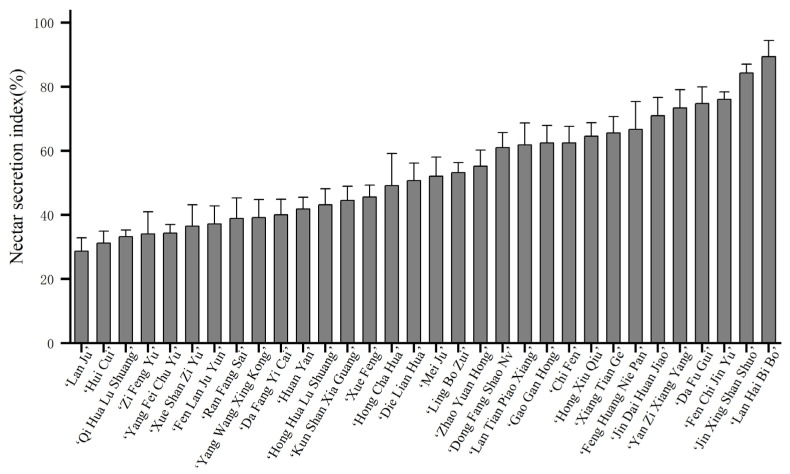
Nectar secretion index of flower buds of 32 *P. lactiflora* cultivars. Note: Data shown in the graphs are means ± standard deviation.

No nectarless flower buds were found in 32 tested cultivars. The nectar secretion index of the flower buds reflected the amount of nectar secretion to a certain extent, and there was notable variation among different cultivars (Figure 5). Among them, ‘Lan Ju’, ‘Hui Cui’, ‘Qi Hua Lu Shuang’, ‘Zi Feng Yu’, ‘Yang Fei Chu Yu’, and ‘Xue Shan Zi Yu’ exhibited lower nectar indices, while ‘Jin Xing Shan Shuo’ and ‘Lan Hai Bi Bo’ exhibited high nectar indices.

**Figure 6:**
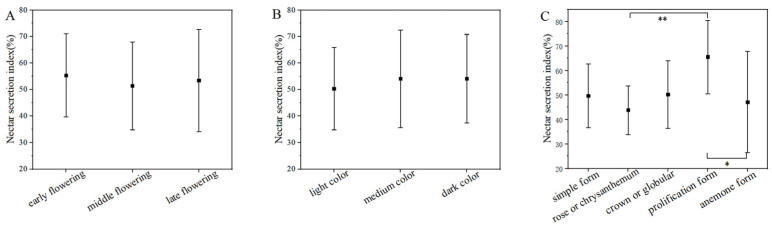
The relationship between nectar secretion index and flowering period, flower color and flower type. Note: The data shown in the figure are mean ± standard deviation. Multiple comparisons of flowering period (**A**), flower color (**B**), and flower type (**C**) with the nectar index were performed by the LSD method, * *p* < 0.05, ** *p* < 0.01.

The analysis of the relationship between the nectar index of different cultivars and the flowering period, flower color, and flower type is shown in Figure 6.

The nectar secretion indices of *P. lactiflora* cultivars with varying flowering periods ranged from 51.40% to 55.38% (see Figure 6A). However, there were no significant differences among the flowering periods. Cultivars within the same flowering period also displayed significant variation in their indices. For instance, the early-flowering cultivar ‘Da Fu Gui’ exhibited an index of 74.67%, while ‘Qi Hua Lu Shuang’ had an index of only 33.11%. The mid-season cultivar ‘Jin Xing Shan Shuo’ exhibited an index of 84.22%, while ‘Lan Ju’ had an index of only 28.67%. Among the late-season cultivars, ‘Lan Hai Bi Bo’ reached an index of 89.33%, while ‘Yang Fei Chu Yu’ had an index of only 34.22%. The correlation analysis (Table 2) indicated no significant correlation between the nectar secretion index of cultivars and their flowering period (|*r*| = 0.0088, *p* = 0.631).

In this trial, the 32 cultivars were categorized based on flower color. The average nectar secretion indices for various flower colors ranged from 50.39% to 54.09%, with no significant differences observed (Figure 6B). However, significant variations in nectar secretion indices were noted among cultivars within the same color category. The greatest range of variation was displayed by medium-toned cultivars, with the highest index recorded in ‘Lan Hai Bi Bo’ and the lowest in ‘Lan Ju’—a difference of 60.67%. The correlation analysis (Table 2) showed no significant correlation between the cultivar’s nectar secretion index and its flower color (|*r*| = 0.075, *p* = 0.684).

The nectar secretion index varies among different cultivars of the same flower type, and even within the same cultivar, there is significant variation in the index (Figure 6C). Although correlation analysis (Table 2) indicated no significant relationship between the nectar secretion index and flower type (|*r*| = 0.116, *p* = 0.529), a pattern did emerge. The nectar secretion index of anemone form cultivars ranged from 28.67% to 84.22%, exhibiting considerable variation among cultivars. The nectar secretion indices of prolification form cultivars were the highest among all flower types at 65.49%, while the average indices for rose or chrysanthemum form cultivars were the lowest, at only 43.85%. The nectar secretion index of different flower types was as follows: prolification form cultivars > crown or globular form cultivars > simple form cultivars > anemone form cultivars > rose or chrysanthemum form cultivars. The difference in nectar secretion indices between prolification form cultivars and anemone form cultivars was significant (*p* < 0.05), while the difference for rose or chrysanthemum form cultivars was highly significant (*p* < 0.01).

### 3.3. Nectar Production of Cultivars with Different Nectar Secretion Index at Different Stages

**Figure 7:**
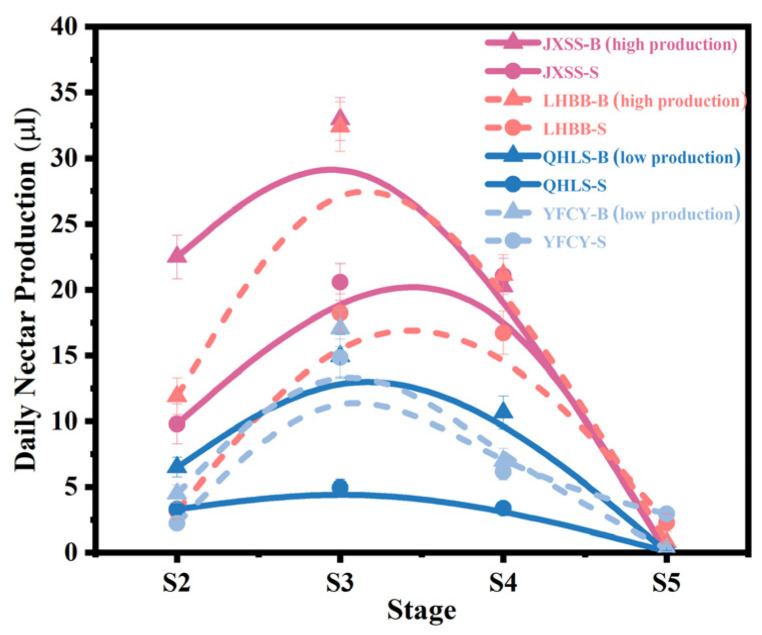
Nectar secretion in *P. lactiflora* cultivars at various growth stages. Note: In the legend, ‘B’ represents bracts, and ‘S’ represents sepals.

Based on the previous results, cultivars with both lower and higher nectar secretion were selected for further quantitative analysis of nectar yield during different secretion phases. The cultivars with lower secretion were ‘Qi Hua Lu Shuang’ and ‘Yang Fei Chu Yu’, while the cultivars with higher secretion were ‘Lan Hai Bi Bo’ and ‘Jin Xing Shan Shuo’, as shown in Figure 7. The daily nectar production of the various cultivars initially increased, then declined across the four developmental stages, peaking during stage S3. Among these cultivars, those with higher nectar secretion indices, such as ‘Jin Xing Shan Shuo’ and ‘Lan Hai Bi Bo’, consistently produced higher nectar yields than those with lower indices, such as ‘Qi Hua Lu Shuang’ and ‘Yang Fei Chu Yu’, from stages S2 to S4. These results further validate the effectiveness of using nectar secretion indices to quickly distinguish between cultivars with differing nectar secretion capacities. Additionally, throughout the nectar secretion period, the volume of nectar in bracts consistently exceeded that in sepals, indicating that bract nectaries contribute more significantly to nectar secretion.

### 3.4. Nectar Sugar Concentration of Cultivars with Different Nectar Yields

**Figure 8:**
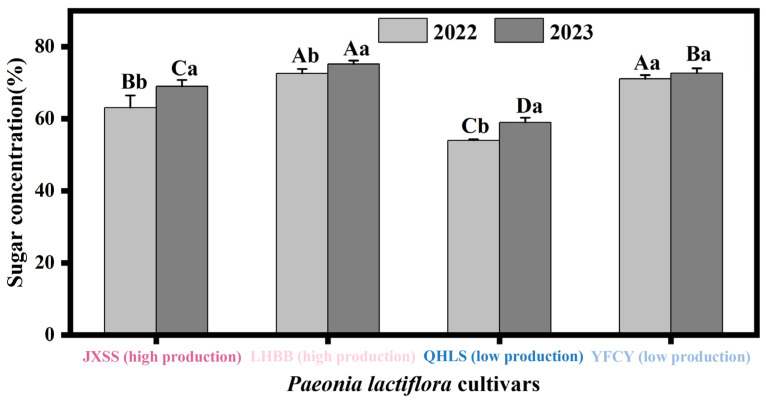
Nectar sugar concentration in two consecutive years of nectar-producing cultivars with differences. Note: Different uppercase letters indicate significant differences among cultivars within the same year (*p* < 0.05). Different lowercase letters indicate significant differences across years for the same cultivar (*p* < 0.05).

During the S3 stage of their respective flower buds, the nectar-rich cultivars ‘Jin Xing Shan Shuo’ and ‘Lan Hai Bi Bo’ were selected alongside the lower-nectar-producing cultivars ‘Qi Hua Lu Shuang’ and ‘Yang Fei Chu Yu’ to test nectar sugar concentration. The results are presented in Figure 8. The nectar sugar concentrations of the high-nectar cultivars ‘Lan Hai Bi Bo’ and ‘Jin Xing Shan Shuo’ were 73.87% and 66.05%, respectively, while those of the low-nectar cultivars ‘Qi Hua Lu Shuang’ and ‘Yang Fei Chu Yu’ were 56.46% and 71.89%, respectively. Among the four cultivars in the same year, sugar concentration followed the order ‘Lan Hai Bi Bo’ > ‘Yang Fei Chu Yu’ > ‘Jin Xing Shan Shuo’ > ‘Qi Hua Lu Shuang’, showing no consistency with nectar secretion levels. Additionally, variations were observed between years. In 2022, there was no significant difference in nectar sugar concentration between ‘Lan Hai Bi Bo’ and ‘Yang Fei Chu Yu’, while both differed significantly from ‘Jin Xing Shan Shuo’ and ‘Qi Hua Lu Shuang’. In 2023, the sugar concentrations of the four cultivars differed significantly from one another and were all higher than those in 2022. Of these, the sugar concentration of ‘Yang Fei Chu Yu’ did not differ significantly between the two years, while the other cultivars showed significant differences. Nectar sugar concentration exhibited no correlation with nectar secretion levels among the cultivars.

### 3.5. Sugar Components of P. lactiflora Nectar

The nectar from two cultivars, ‘Lan Hai Bi Bo’ (high nectar production) and ‘Yang Fei Chu Yu’ (low nectar production), was analyzed to determine its sugar composition. The concentrations of 32 distinct sugars in the nectar of these two cultivars were analyzed. Both cultivars contained 23 identical components, while nine additional components were not detected. The results are presented in Table 3. *P. lactiflora* nectar is classified as belonging to the sucrose-dominant nectar category. Metabolites exhibiting fold changes of at least 2 and no more than 0.5 were classified as differential metabolites between the two cultivars. The results indicate that no significant differences were found among the other sugar components, with the exception of D-ribose and D-xylulose.

### 3.6. Ultrastructural Differences in the Epidermal Cells Between Nectar-Secreting and Non-Nectar-Secreting Regions of Bracts During Development

**Figure 9:**
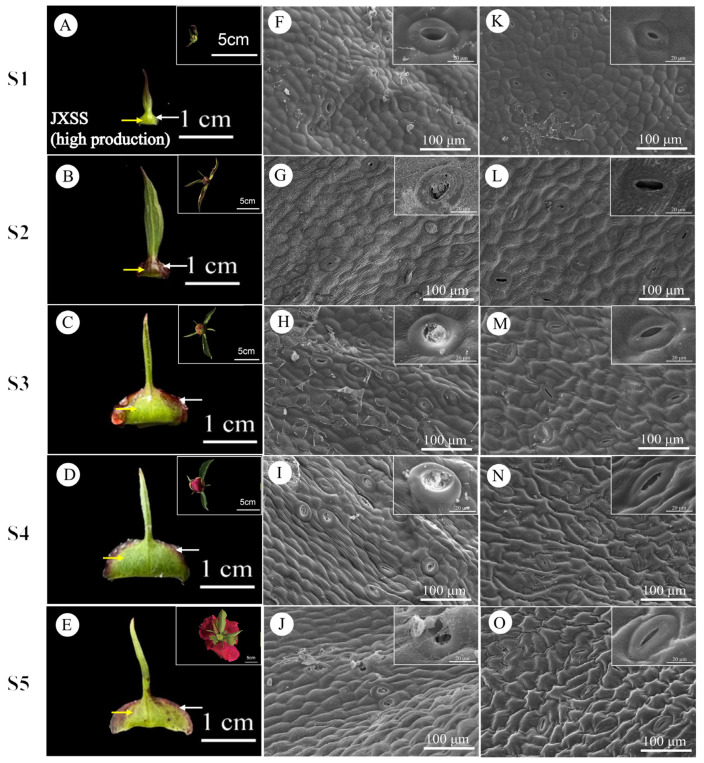
Macroscopic and surface morphology of different regions of the bracts at different stages of secretion in JXSS (high production). (**A**–**E**) employ white arrows to indicate the nectar-secreting areas on the JXSS bracts, while yellow arrows denote the non-nectar-secreting areas. (**F**–**J**) illustrate the microstructure of the swollen edge at the base of the bract (the nectar-secreting area). (**K**–**O**) depict the microstructure of the non-edge area of the bract (the non-nectar-secreting area). (**A**–**E**) Inset shows flower buds from the same period; (**F**–**O**) inset shows an enlarged view of stomata.

**Figure 10:**
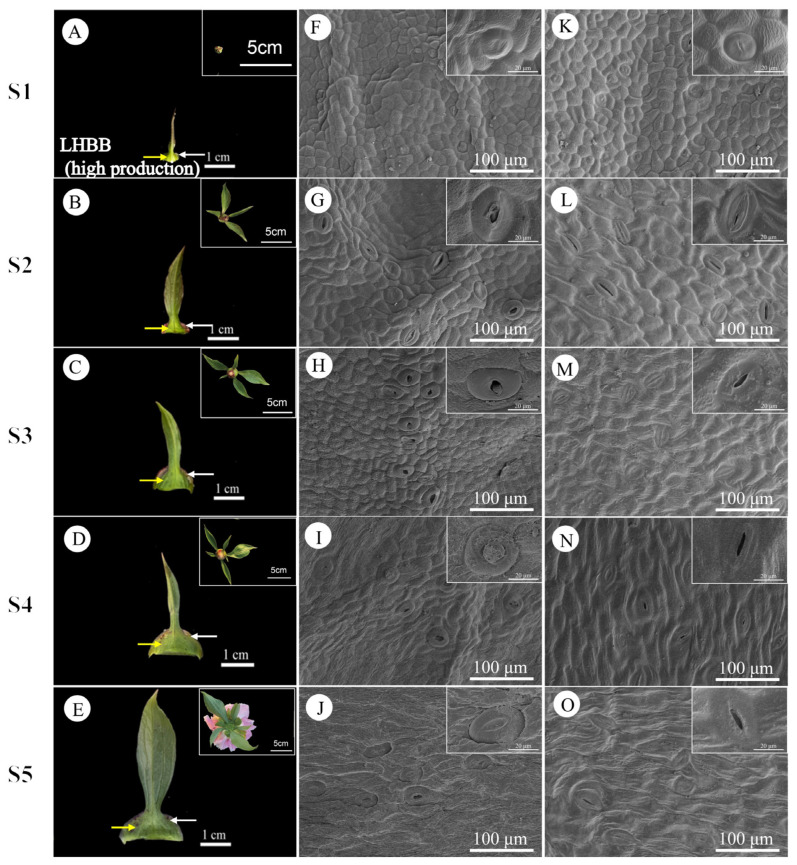
Macroscopic and surface morphology of different regions of the bracts at different stages of secretion in LHBB (high production). (**A**–**E**) employ white arrows to indicate the nectar-secreting areas on the LHBB bracts, while yellow arrows denote the non-nectar-secreting areas. (**F**–**J**) illustrate the microstructure of the swollen edge at the base of the bract (the nectar-secreting area). (**K**–**O**) depict the microstructure of the non-edge area of the bract (the non-nectar-secreting area). (**A**–**E**) Inset shows flower buds from the same period; (**F**–**O**) inset shows an enlarged view of stomata.

**Figure 11:**
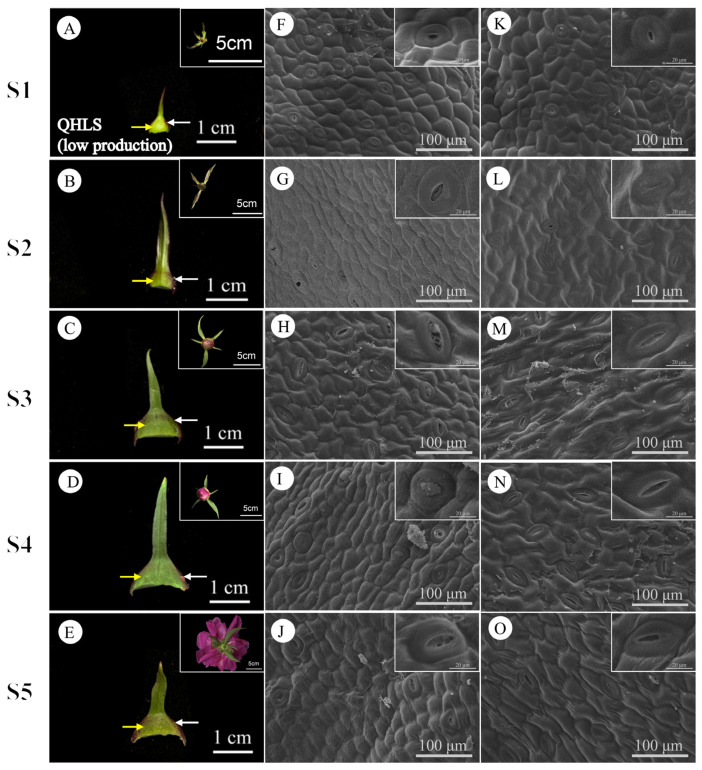
Macroscopic and surface morphology of different regions of the bracts at different stages of secretion in QHLS (low production). (**A**–**E**) employ white arrows to indicate the nectar-secreting areas on the QHLS bracts, while yellow arrows denote the non-nectar-secreting areas. (**F**–**J**) illustrate the microstructure of the swollen edge at the base of the bract (the nectar-secreting area). (**K**–**O**) depict the microstructure of the non-edge area of the bract (the non-nectar-secreting area). (**A**–**E**) Inset shows flower buds from the same period; (**F**–**O**) inset shows an enlarged view of stomata.

**Figure 12:**
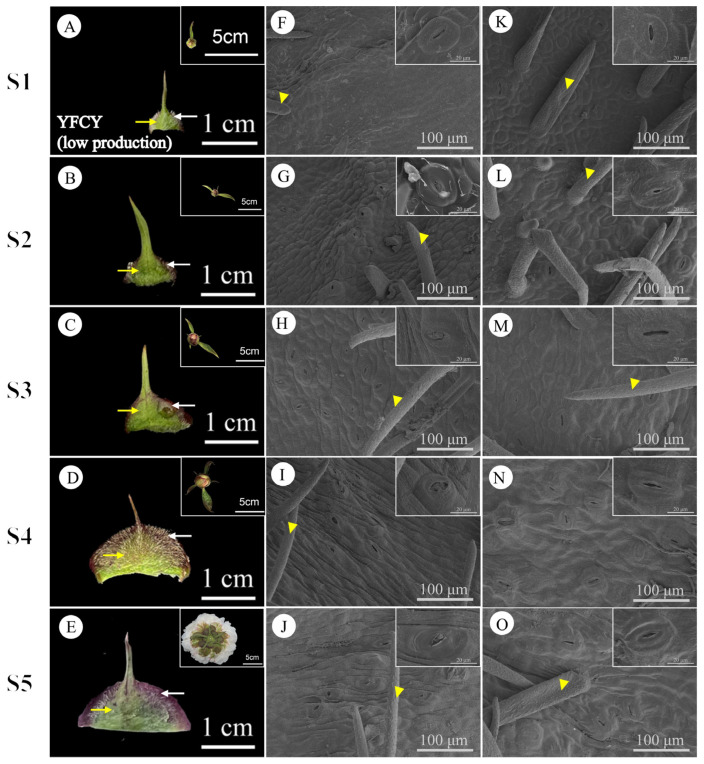
Macroscopic and surface morphology of different regions of the bracts at different stages of secretion in YFCY (low production). (**A**–**E**) employ white arrows to indicate the nectar-secreting areas on the YFCY bracts, while yellow arrows denote the non-nectar-secreting areas. (**F**–**J**) illustrate the microstructure of the swollen edge at the base of the bract (the nectar-secreting area). (**K**–**O**) depict the microstructure of the non-edge area of the bract (the non-nectar-secreting area). Yellow triangles indicate non-glandular trichomes. (**A**–**E**) Inset shows flower buds from the same period; (**F**–**O**) inset shows an enlarged view of stomata.

During stages S1 to S5, the daily nectar production of bracts and sepals displayed consistent patterns. Bracts consistently secreted more nectar than sepals, indicating that bract nectaries play a more significant role in nectar production. Therefore, the subsequent analysis and discussion in this paper will primarily focus on bracts. The external epidermal microstructure of the bracts during nectar secretion was examined in four cultivars with varying nectar yields. Similar microstructural changes were observed in both nectar-secreting and non-secreting areas across all cultivars. Figure 9 and Figure 10 illustrate the high-yield cultivars ‘Jin Xing Shan Shuo’ and ‘Lan Hai Bi Bo’; Figure 11 and Figure 12 illustrate the low-yield cultivars ‘Qi Hua Lu Shuang’ and ‘Yang Fei Chu Yu’.

Over the course of nectar secretion, no differential changes were observed in the stomata and epidermal cells at the bract secretion sites among the four cultivars. In S1, stomata were newly formed, and epidermal cells were flat and irregularly shaped (F in Figure 9, Figure 10, Figure 11 and Figure 12). In S2, the stomata were open and raised on the surface of the epidermis, often distributed above the vascular tissue. Nectar secretion was evident in the stomatal apparatus, and epidermal cells appeared plump and polygonal with reticulate ornamentation (G in Figure 9, Figure 10, Figure 11 and Figure 12). In S3, numerous specialized stomata appeared, with the majority of the stomata in an open state exhibiting a large aperture. A clear honeydew secretion could be observed within the stomatal channels. Epidermal cells were rough with pronounced reticulate ornamentation (H in Figure 9, Figure 10, Figure 11 and Figure 12). In S4, the stomata remained open, but most nectariferous stomata were nearly clogged by granular material, likely crystallized nectar (I in Figure 9, Figure 10, Figure 11 and Figure 12). In S5, most stomata were extruded and deformed, with negligible nectar secretion, and the epidermal cells were aged and atrophied (J in Figure 9, Figure 10, Figure 11 and Figure 12).

Over the course of nectar secretion, no differential changes were observed in the stomata and epidermal cells at the bract no-secretion sites among the four cultivars. The changes in the stomata and epidermal cells in non-marginal bracts were as follows: Stomata were at the early stage of formation in S1, and epidermal cells exhibited irregular morphology (K in Figure 9, Figure 10, Figure 11 and Figure 12). From S2 to S4, the stomata showed two states of opening and closing, with no obvious changes in morphology and no secretion. The stomata were flat with small openings, while the epidermal cells showed no notable alteration (L–N in Figure 9, Figure 10, Figure 11 and Figure 12). Lastly, in S5, the stomata were compressed and deformed, while the epidermal cells were shrunken (O in Figure 9, Figure 10, Figure 11 and Figure 12). Stomatal developmental dynamics differed between the non-nectar and nectar sides.

### 3.7. Stomatal Characteristics of the Nectary Epidermis at Bracts and Sepals During Stage 3

The surface-stomata-related indices of nectar-secreting parts of cultivars with differing nectar secretion levels were analyzed. Four of these parameters, namely stomatal length, stomatal width, stomatal area, and stomatal openness, did not meet the assumptions required for analysis of variance (ANOVA). Consequently, non-parametric tests were employed. One-way ANOVA was performed to evaluate the stomatal density and the percentage of nectar-secreting stomata, while independent-samples t-tests were used to analyze the stomatal-related indices of bracts and sepals during the S3 period. The results are summarized in Table 4. The high-nectar-production cultivars exhibited a significantly greater stomatal width at the bract margins compared to the low-nectar-production cultivars. The difference between the length and width of the sepal edge stomata in the high-nectar-production cultivars and the low-nectar-production cultivar ‘Yang Fei Chu Yu’ was not significant. Furthermore, the openness of the bract and sepal stomata in the high-nectar-yield cultivars ranged from 6.17 to 15.16 µm and from 5.18 to 14.82 µm, respectively, which were 82.10% and 52.84% higher than those in the low-nectar-production cultivars. The percentage of bract and sepal nectar-secreting stomata in the high-nectar-producing cultivars ranged from 45.45% to 80.00% and from 44.44% to 81.82%, respectively, whereas these percentages were significantly lower by 181.28% and 149.01% in the low-nectar-producing cultivars.

Analyses of surface stomatal correlation indices at the edges of the bracts and sepals of the same cultivars revealed that the indices at the edges of the bracts of ‘Lan Hai Bi Bo’ were significantly higher than those at the edges of the sepals. The stomatal area and openness of the bract margin of ‘Jin Xing Shan Shuo’ were significantly higher than those of the sepals, while the stomatal density and percentage of nectar-secreting stomata were significantly lower than those of the sepal margins. The difference in stomatal length and width between the two parts was not statistically significant. The width and area of stomata at the edge of the bracts of ‘Yang Fei Chu Yu’ were significantly lower than those at the edge of sepals. In contrast, the density of stomata was significantly higher at the edge of bracts compared to the edge of sepals. The remaining indices did not show statistically significant differences between these two parts. Similarly, the length and area of stomata at the edge of bracts of ‘Qi Hua Lu Shuang’ were significantly higher than those at the edge of sepals. The remaining indices did not exhibit statistically significant differences.

The results of the correlation analysis between the nectar secretion index and the surface stomatal index at the nectar sites of *P. lactiflora* cultivars with varying nectar production levels are presented in Table 5. The nectar secretion index was significantly positively correlated with bract margin stomatal width (*r* = 0.711, *p* < 0.01), stomatal aperture (*r* = 0.711, *p* < 0.01), and the percentage of nectar-secreting stomata (*r* = 0.725, *p* < 0.01). Furthermore, the nectar secretion index was also significantly positively correlated with sepal margin stomatal aperture (*r* = 0.666, *p* < 0.05) and the percentage of nectar-producing stomata (*r* = 0.725, *p* < 0.01). Stomatal length, area, and density showed no correlation with nectar secretion in *P. lactiflora*.

### 3.8. Anatomical Structure of the Nectary Tissue Within the Bracts

**Figure 13:**
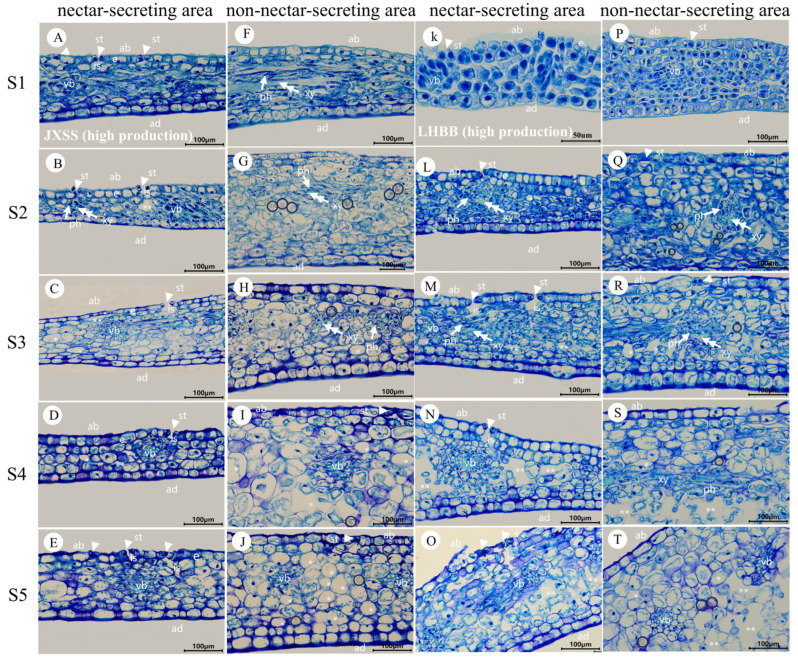
Anatomical structure regions of the bracts at different stages of secretion in JXSS and LHBB (high production). (**A**,**F**), (**B**,**G**), (**C**,**H**), (**D**,**I**), and (**E**,**J**) show bracts at S1, S2, S3, S4, and S5 of JXSS, respectively. (**A**–**E**) illustrate the anatomical structure of the swollen edge at the base of the JXSS bract (the nectar-secreting area). (**F**–**J**) depict the anatomical structure of the non-edge area of the bract (the non-nectar-secreting area). (**K**,**P**), (**L**,**Q**), (**M**,**R**), (**N**,**S**), and (**O**,**T**) show bracts at S1, S2, S3, S4, and S5 of LHBB, respectively. (**K**–**O**) illustrate the anatomical structure of the swollen edge at the base of the bract (the nectar-secreting area). (**P**–**T**) depict the anatomical structure of the non-edge area of the bract (the non-nectar-secreting area). e: secretory epidermis; ab: abaxial surface; ad: adaxial surface; vb: vascular bundle; ph: phloem; xy: xylem; is: substomatal chamber. Triangular arrows indicate stomata; double arrowheads indicate the xylem; single arrowheads indicate the phloem; ‘*’ indicates massive vacuolated cells; ‘**’ indicates intercellular spaces; and circles indicate calcium oxalate crystals.

**Figure 14:**
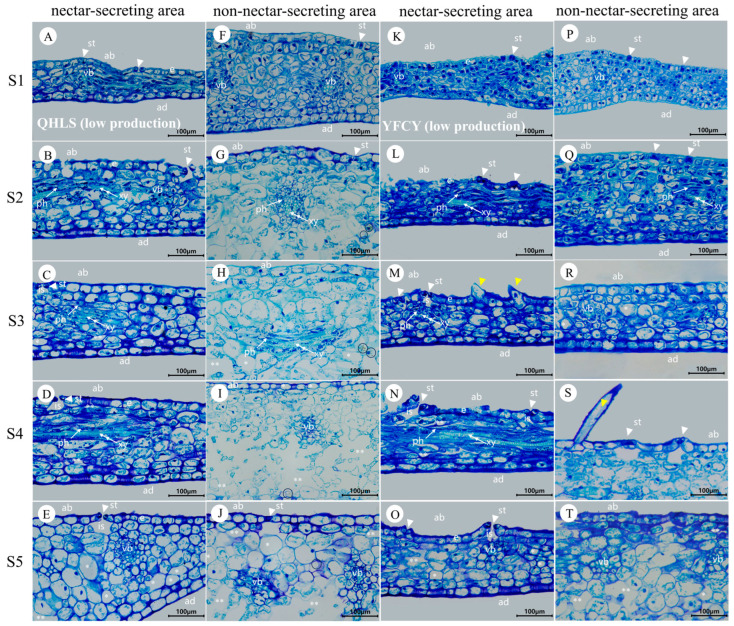
Anatomical structure regions of the bracts at different stages of secretion in QHLS and YFCY (low production). (**A**,**F**), (**B**,**G**), (**C**,**H**), (**D**,**I**), and (**E**,**J**) show bracts at S1, S2, S3, S4, and S5 of QHLS, respectively. (**A**–**E**) illustrate the anatomical structure of the swollen edge at the base of the QHLS bract (the nectar-secreting area). (**F**–**J**) depict the anatomical structure of the non-edge area of the bract (the non-nectar-secreting area). (**K**,**P**), (**L**,**Q**), (**M**,**R**), (**N**,**S**), and (**O**,**T**) show bracts at S1, S2, S3, S4, and S5 of YFCY, respectively. (**K**–**O**) illustrate the anatomical structure of the swollen edge at the base of the bract (the nectar-secreting area). (**P**–**T**) depict the anatomical structure of the non-edge area of the bract (the non-nectar-secreting area). e: secretory epidermis; ab: abaxial surface; ad: adaxial surface; vb: vascular bundle; ph: phloem; xy: xylem; is: substomatal chamber. Triangular arrows indicate stomata; double arrowheads indicate the xylem; single arrowheads indicate the phloem; ‘*’ indicates massive vacuolated cells; ‘**’ indicates intercellular spaces; and circles indicate calcium oxalate crystals. Yellow triangles indicate non-glandular trichomes.

Figure 13 and Figure 14 illustrate the results for the bract nectaries of the high-nectar-producing cultivars ‘Jin Xing Shan Shuo’ and ‘Lan Hai Bi Bo’, as well as the low-nectar-producing cultivars ‘Qi Hua Lu Shuang’ and ‘Yang Fei Chu Yu’.

The nectaries of the high-nectar cultivars ‘Jin Xing Shan Shuo’ and ‘Lan Hai Bi Bo’ exhibited larger stomata apertures (see Figure 13C,M). However, no significant structural differences were found in the nectar-producing regions of the bracts among cultivars with varying nectar yields (see Figure 13 and Figure 14, panels A–E and K–O). The apical surface of the enlarged basal region of the bract bears numerous stomata that are distributed along its margin. This area constitutes the nectary epidermis, composed of a single layer of cells. Vascular bundles were often distributed under the stomata, which were 0–1 layer of cells away from the vascular bundles, and the epidermis of the adaxial surface was without stomata. During the S1–S5 period, the anatomical structure of the nectar-producing regions underwent dynamic changes:

S1—stomata were incompletely differentiated; most of them were not open, with higher cytoplasmic density and larger nuclei, and did not produce nectar at that time (Figure 13 and Figure 14A,K);

S2—stomata open, amphicribral vascular (Figure 13 and Figure 14B,L);

S3—the substomatal chambers were clearly visible, the stomatal apertures were wide, and intercellular spaces were present (Figure 13 and Figure 14C,M);

S4—the cell was partially vacuolated, and the nucleus was adhered to the wall (Figure 13 and Figure 14D,N);

S5—the cell vacuolization was obvious, and the modified stomata remained open (Figure 13 and Figure 14E,O).

Non-nectar-producing regions (F–J and P–T in Figure 13 and Figure 14): The sparse stomatal distribution on non-nectariferous parts or normal stomatal morphology in the epidermis of the abaxial surface and vascular bundles do not correspond to the distribution under the stomata, and the upper epidermis had 2–5 layers of cells at a distance from the vascular bundles. The cells were packed tightly during S1 and S2 (Figure 13 and Figure 14F,P,Q), and the parenchyma cell showed severe vacuolation, sparse and deformed after the S3 stage (Figure 13 and Figure 14H,R). The distribution of calcium oxalate crystals in ‘Lan Hai Bi Bo’ and ’Jin Xing Shan Shuo’ with high nectar secretion and ‘Qi Hua Lu Shuang’ with low nectar secretion was common in stages S2–S5 (Figure 13G–J,Q–T and Figure 14G–J), but the epidermis of ‘Yang Fei Chu Yu’ with low nectar secretion is distributed in non-glandular trichomes (Figure 14S), and calcium oxalate crystals were occasionally found in the tissues (Figure 14Q). In summary, nectaries are classified as structural nectaries, composed of a secretory epidermis with numerous modified stomata, nectar-producing tissue with dense cytoplasm, and vascular bundles closely associated with the stomata.

### 3.9. The Dynamics of Starch During Nectar Secretion

**Figure 15:**
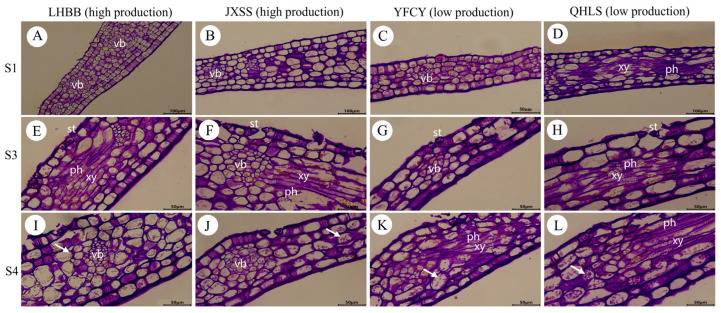
Dynamic changes of starch in bracts of four *P. lactiflora* cultivars during nectar secretion. Note: (**A**,**E**,**I**) Sections of bract margin tissue from LHBB; (**B**,**F**,**J**) sections of bract margin tissue from JXSS; (**C**,**G**,**K**) sections of bract margin tissue from YFCY; (**D**,**H**,**L**) sections of bract margin tissue from QHLS. Arrows indicate starch granules. st: stomata; vb: vascular bundle; ph: phloem; xy: xylem.

To investigate the origin of nectar, PAS staining was conducted on the nectar-secreting regions of bracts from four cultivars with varying nectar yields. PAS staining highlights polysaccharides in red (see Figure 15). The results indicated that, during the S1 stage, most stomata remained closed across all four cultivars. The vascular bundles, often located beneath the stomata, formed a reticulated network that established extensive pre-nectar transport pathways without starch accumulation (see Figure 15A–D). During the S3 stage, darker-stained sieve tubes were observed surrounding the vessels, acting as the primary source of nectar. No starch storage was found in the nectar-secreting regions, and the sub-pore cavities were enlarged (Figure 15E–H). At the S4 stage, intercellular spaces became apparent between cells, and the guard cells lost their ability to close, resulting in the stomata remaining open (Figure 15I–L). In summary, the nectar glands of all four cultivars exhibited no starch accumulation prior to nectar secretion, indicating that *P. lactiflora* nectar does not originate from starch reserves.

### 3.10. Photosynthetic Pigment Content During Nectar Production in Different Cultivars

**Figure 16:**
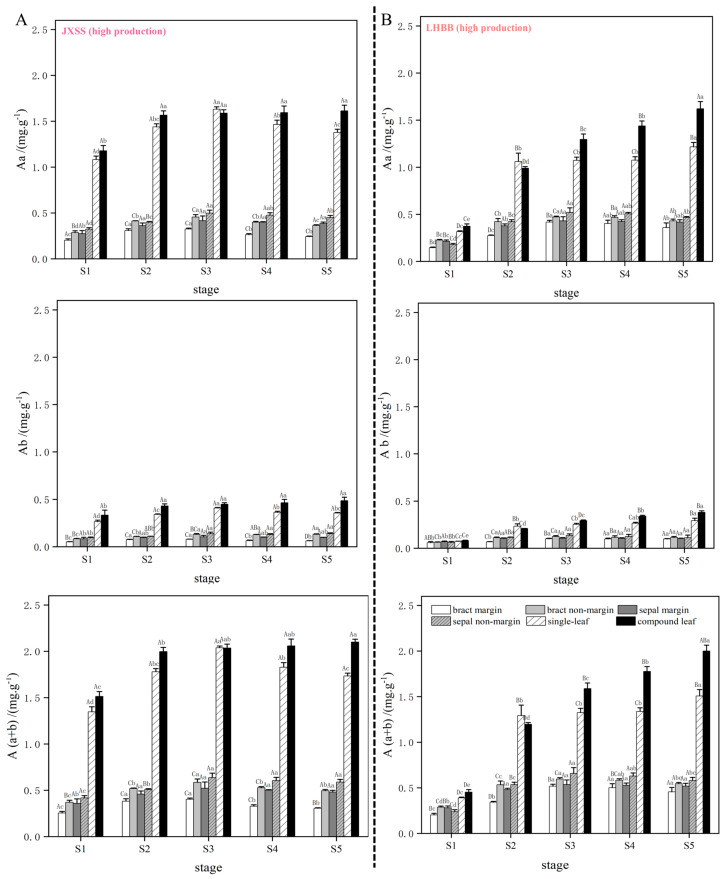
Content of photosynthetic pigments in different parts of *P. lactiflora* high-production cultivars during nectar secretion. Note: (**A**,**B**) illustrate the dynamic changes in the content of chlorophyll a, chlorophyll b, and total chlorophyll during the nectar secretion process of JXSS and LHBB. Uppercase letters indicate differences between cultivars at the same stage; lowercase letters indicate differences in the same cultivar at different stages.

**Figure 17:**
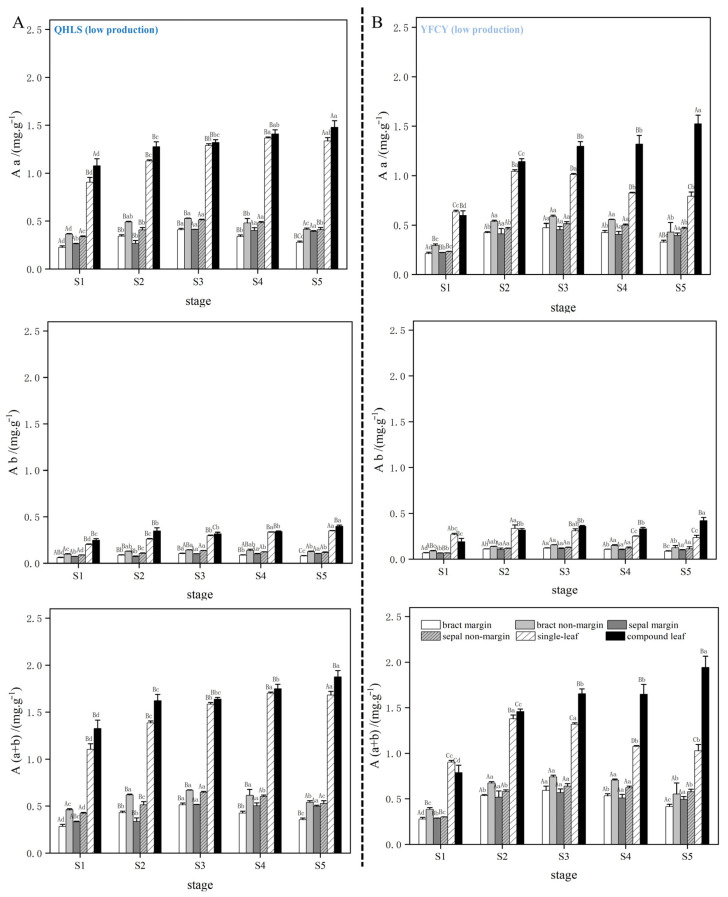
Content of photosynthetic pigments in different parts of *P. lactiflora* low-production cultivars during nectar secretion. Note: (**A**,**B**) illustrate the dynamic changes in chlorophyll a, chlorophyll b, and total chlorophyll content during nectary secretion in the QHLS and YFCY cultivars, respectively. Capital letters signify differences between cultivars at the same developmental stage, while lowercase letters denote variations within the same cultivar across different developmental stages.

The results indicated that the levels of chlorophyll a and (a + b) in the bracts and sepals (in both nectary and non-nectary regions) of all four cultivars displayed an initial increase followed by a decrease as nectar secretion progressed. At the S3 stage (peak nectar secretion), levels stabilized or significantly declined, while chlorophyll b exhibited minimal variation. No significant differences in chlorophyll content were found among cultivars with varying nectar yields. The photosynthetic pigment content in the simple and compound leaves of the four cultivars was higher than that in the bracts and sepals, with both showing a gradual increase as nectar secretion progressed. However, the high-nectar cultivars ‘Lan Hai Bi Bo’ and ‘Jin Xing Shan Shuo’ displayed similar patterns to the low-nectar cultivars ‘Yang Fei Chu Yu’ and ‘Qi Hua Lu Shuang’ (see Figure 16 and Figure 17), indicating no significant differences in nectar secretion among the cultivars.

### 3.11. Chlorophyll Fluorescence Parameters Fv/Fm of Each Part of Four P. lactiflora Cultivars in Period S3

**Figure 18:**
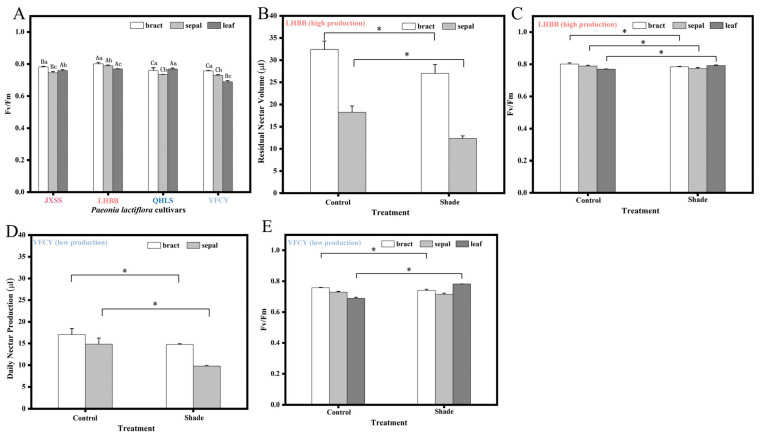
Fv/Fm in various parts of *P. lactiflora* and the response of nectar production and Fv/Fm to shading. Note: (**A**) Fv/Fm ratios for various parts of four cultivars during S3; (**B**) daily nectar production for LHBB under shading treatment; (**C**) Fv/Fm ratio for LHBB under shading treatment; (**D**) daily nectar production under shading treatment for YFCY; (**E**) Fv/Fm ratio under shading treatment for YFCY. Uppercase letters are differences between cultivars of the same position; lowercase letters are differences between different positions of the same cultivar.

Since nectar does not originate from stored starch, it is likely derived from photosynthesis. Measurements of Fv/Fm across different plant parts during the S3 stage for cultivars with varying levels of nectar secretion revealed the following results (see Figure 18A). The two cultivars with high nectar secretion exhibited significantly higher Fv/Fm values in both the bracts and sepals compared to the two cultivars with low nectar secretion. The Fv/Fm values of the bracts in all four cultivars were higher than those of the sepals and compound leaves. This indicates that photosystem II (PSII) in the bracts is more efficient at utilizing light energy than PSII in the leaves. After shading treatment, both the high-nectar-producing cultivar ‘Lan Hai Bi Bo’ and the low-nectar-producing cultivar ‘Yang Fei Chu Yu’ exhibited decreased daily nectar secretion in the bracts and sepals (Figure 18B), while Fv/Fm values in both the bracts and sepals also showed a decreasing trend (Figure 18C). The low-nectar-producing cultivar ‘Yang Fei Chu Yu’ demonstrated the same trend (Figure 18D,E). This suggests a correlation between photosynthetic capacity and nectar secretion.

### 3.12. Response of P. lactiflora Nectar Secretion to External Liquid Spraying

**Figure 19:**
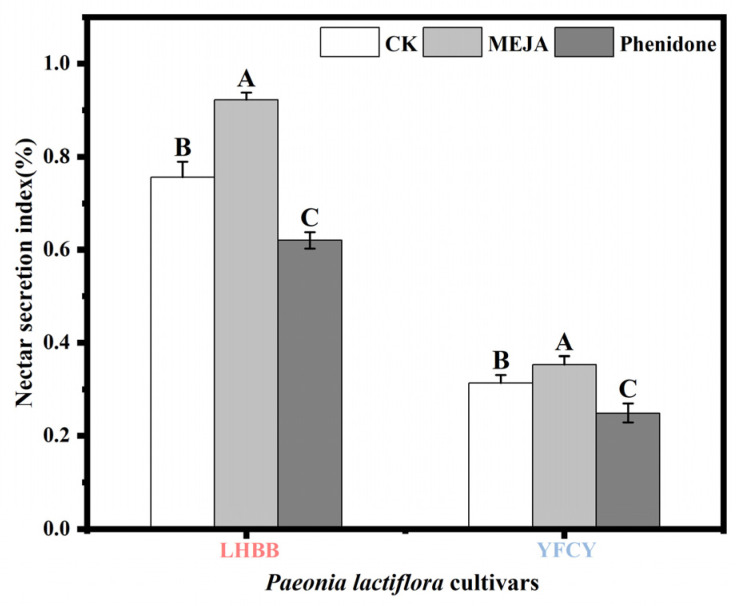
Nectar secretion index of LHBB and YFCY after external liquid spraying. Note: Different uppercase letters indicate significant differences among different treatments within the same cultivar (*p* < 0.05).

Figure 19 presents the results of external spraying of flower buds during the S2 stage and the corresponding recording of nectar secretion during the S3 stage. Treatment with methyl jasmonate (MEJA) significantly increased the nectar secretion index for the ‘Lan Hai Bi Bo’ and ‘Yang Fei Chu Yu’ flower buds, thus promoting nectar secretion. In contrast, treatment with the inhibitor phenidone significantly reduced the nectar secretion index in the buds of the ‘Lan Hai Bi Bo’ and ‘Yang Fei Chu Yu’ cultivars, thus inhibiting nectar secretion.

## 4. Discussion

### 4.1. Determination of P. lactiflora Nectar Yield and Nectar Secretion Characteristics

Different cultivars of cultivated plants exhibit variations in nectar secretion characteristics, including timing, quantity, concentration, and composition [42]. *Paeonia lactiflora* possesses multiple nectar-producing sites, a high nectar yield, and viscous nectar. This complicates the accurate and rapid measurement of nectar quantities across its various cultivars. Grading its nectar secretion levels represents an effective method for the rapid assessment of nectar production. Nectar secretion sites and quantities in *P. lactiflora* undergo dynamic changes throughout growth, primarily occurring at the basal margins of bracts and the edges of sepals on the apical surface. The primary site of nectar secretion shifts centripetally during development, from the bracts to the sepals. This process is structurally analogous to that observed in daylilies (*Hemerocallis citrina*), where the nectaries transition from the sepals prior to flowering to the pistil nectaries after full bloom [6].

Nectar primarily consists of sugar solutions, with sucrose, fructose, and glucose as the most common components [43]. However, the predominant sugar composition varies among different species. For example, nectar from *Arabidopsis thaliana*, *Leptospermum*, and *Polemonium caeruleum* is predominantly composed of hexoses [44,45,46], whereas nectar from *Ananas ananassoides*, *Nicotiana tabacum*, and *Cucurbita pepo* is predominantly composed of sucrose [47,48,49]. Oligosaccharides, such as stachyose and raffinose, have also been detected in the nectar of certain plants [50]. The results of this study indicate that *P. lactiflora* nectar contains significantly higher levels of sucrose compared to other sugars, thereby classifying it as sucrose-dominant. Furthermore, its nectar contains elevated levels of sugar alcohols, including inositol. These compounds are typically closely associated with nectar secretion activity and are more abundant both prior to and during the secretion phase [51]. This finding suggests that these compounds may play a significant role in the synthesis and secretion of *P. lactiflora* nectar.

Field measurements frequently utilize handheld refractometers to assess the sugar content of nectar [52]. Reported nectar sugar concentrations for the majority of plants range from 6% to 40% [28,53]. In this study, *P. lactiflora* nectar was found to have a sugar content ranging from 53.50% to 76.85%, indicating a notably high concentration of sugar.

### 4.2. Nectar Structure of the P. lactiflora

In recent years, nectaries located on floral organs but not involved in pollination have been classified as extrafloral nectaries [6,7,8,9,10]. These nectaries display considerable morphological diversity. For instance, those of *Clerodendrum chinense* appear as disc-shaped protrusions at the leaf base [51], whereas the extrafloral nectaries of *Brassica juncea* lack typical glandular structures and can only be identified by the presence of nectar [54]. This study reveals that *P. lactiflora* nectaries are extrafloral, situated on the outer margins of bracts and sepals. Structurally, this nectary morphology, which shows no distinct external protrusion and is level with the epidermal surface, resembles the nectary structures reported in *Liriodendron tulipifera* and *Anemone cathayensis* [34,55].

*Paeonia lactiflora* nectaries are structural nectaries featuring specialized stomata that are distributed across their epidermis. Nectaries that secrete nectar through modified stomata typically display the following characteristics: well-developed intercellular spaces within the nectar-producing tissue, modified stomata that have lost their capacity to close, and enlarged stomatal chambers. This facilitates the accumulation of nectar in the stomatal chambers via the intercellular spaces prior to secretion through the stomata [56,57,58]. *Paeonia lactiflora* nectaries display these characteristics, with stomata protruding from the nectary epidermis. Vascular bundles are frequently located beneath the specialized stomata of *P. lactiflora* nectaries, separated by just one or two cell layers. Both *Echinacea purpurea* and *Anthurium andreanum* secrete nectar through specialized stomata. The nectary anatomy of *E. purpurea* resembles that of *P. lactiflora* [59]. However, unlike the anatomical findings observed in *A. andreanum* petals, the vascular bundles in purple coneflower terminate at the boundary of the nectar-producing tissue [60]. The presence of vascular bundles adjacent to stomata may enhance the supply of water and sugars necessary for nectary production [61]. Research indicates that the efficiency of nectary secretion relies on the presence or proximity of xylem and phloem [5]. Consequently, the close association between modified epidermal stomata and vascular bundles in *P. lactiflora* nectaries accounts for the substantial nectar secretion capacity.

Research indicates that nectaries possessing stomata secrete more nectar than those lacking them [62]. This may elucidate why *P. lactiflora* is capable of producing such large quantities of nectar. However, findings regarding the effects of stomatal density and area on nectar yield are inconsistent. Some studies demonstrate no clear correlation between nectar production and stomatal density [63]. Nevertheless, *Prunus laurocerasus* ‘Schipkaensis’ exhibits a high volume and concentration of nectar secretion, accompanied by elevated stomatal density and area [46]. This study demonstrates that the volume of *P. lactiflora* nectar secretion significantly correlates with the width of bract nectary stomata, stomatal aperture, and the percentage of nectar-secreting stomata. Conversely, no correlation was identified with stomatal length, stomatal area, or stomatal density. It was hypothesized that large stomatal apertures and a higher percentage of nectar-secreting stomata could induce *P. lactiflora* to secrete more nectar.

### 4.3. Nectar Source of P. lactiflora

Current research indicates that nectar secretion primarily occurs via two pathways. One pathway originates from phloem transport and typically yields larger quantities over shorter durations [64]. The other pathway arises directly from photosynthesis, and nectaries generally lack vascular tissue, resulting in smaller quantities over extended periods [65]. *Paeonia lactiflora* nectar secretion displays characteristics of both high volume and prolonged duration, suggesting that its nectar may derive from photosynthesis. Nevertheless, its nectaries also contain vascular tissue, which suggests that *P. lactiflora* nectar may originate simultaneously from phloem transport—a conclusion supported by the high sucrose concentration observed in its nectar.

Moreover, the absence of starch accumulation during the initial phase of nectar secretion in *P. lactiflora* suggests that nectar does not derive from starch hydrolysis, thereby confirming its classification as a non-starch storage nectary [66]. The concept of green nectaries was initially proposed by Schnepf in 1964 [67]. Photosynthesis occurring in green nectaries significantly contributes to sugar secretion. Reported plant structures, such as pedicels, receptacles, sepals, ovaries, and adjacent leaves, frequently participate in the production of photosynthetic nectar sugars [68,69]. Moreover, research conducted by Cawoy et al. [70] demonstrated that light exposure can directly influence reproductive structures, thereby regulating nectar secretion.

The dynamics of *P. lactiflora* nectar production indicate that nectar secretion commences when the bracts and sepals emerge. Subsequently, as the flower buds enlarge, the bracts and sepals growing beneath them become shaded, resulting in reduced nectar secretion. This suggests that photosynthesis occurring in the bracts and sepals may be the primary source of its nectar. This study found consistent changes in the content of chlorophyll a and chlorophyll b (Chla) within the bracts and sepals of *P. lactiflora* flower buds during nectar secretion. Additionally, variations in the Fv/Fm ratios were observed across different cultivars. These findings suggest that these structures possess photosynthetic potential and may be related to sources of nectar production.

Green nectaries are often referenced in the literature, prompting researchers to speculate that they may utilize photosynthesis to provide the carbohydrates necessary for nectar secretion [33,68]. However, quantitative data regarding the photosynthetic capacity of nectary-bearing tissues remain limited. The concentration of photosynthetic pigments in leaves is a crucial factor that determines a plant’s photosynthetic rate and dry matter production [71]. During the secretion period, the photosynthetic pigment content in both *P. lactiflora* bract and sepal nectaries was greater than that at the pre-secretion and post-secretion stages. Similar findings were observed in the leaf nectaries of double-flowered jasmine [51]. Research confirms that photosynthesis serves as the primary source of nectar sugars in extrafloral nectaries, and that elevated chlorophyll levels during secretion correlate with increased nectar production rates. The chlorophyll fluorescence parameter Fv/Fm reflects a plant’s maximum potential for photosynthetic capacity [68]. In this study, both its nectar production and Fv/Fm values decreased in the bracts and sepals under shaded conditions. Among the four *P. lactiflora* cultivars examined, the Fv/Fm values of the bracts and sepals from cultivars with higher nectar production were significantly greater than those from cultivars with lower nectar production. Moreover, the Fv/Fm value of the bracts from high-nectar-producing cultivars was higher than that of the sepals from low-nectar-producing cultivars. These findings provide preliminary support for the hypothesis that photosynthesis in the green nectaries of *P. lactiflora* could serve as a source for nectar. Further studies with larger sample sizes are needed to statistically validate this relationship.

Previous studies have indicated that phenidone can inhibit the jasmonic acid synthesis pathway by blocking lipoxygenase activity, thus altering the balance between carbon and nitrogen metabolism in plants [39,72,73]. This shifts carbon sources from pathways synthesizing sugars like sucrose to processes that produce nitrogen-containing compounds, such as amino acids. This reallocation of carbon sources significantly inhibits the transport and accumulation of photosynthetic carbon fixation products in nectaries, ultimately leading to reduced nectar secretion. This further supports the notion that the photosynthetic pathway serves as a significant source of carbon for *P. lactiflora* nectar. The decrease in nectar volume observed after the application of phenidone in this study offers additional evidence supporting the photosynthetic pathway as a potential source of carbon for its nectar.

### 4.4. Significance of Nectar Secretion During the Bud Stage of P. lactiflora

Extrafloral nectaries are secretory structures that can initiate indirect defense mechanisms. Currently, more than 100 species of angiosperms produce extrafloral nectar to attract mutualistic animals, such as ants, in exchange for abundant carbohydrates [74,75,76]. Most studies indicate that ants primarily protect plants from herbivory [77]. However, other studies suggest that nectaries may also attract ants to minimize interference with pollination [78]. One intriguing experiment revealed that placing plastic ants on open flowers significantly decreased visits from pollinators [79]. Nectar produced outside the flower serves as a valuable food source for beneficial insects, including ladybirds and spiders. Similar to ants, these insects feed on nectar and assist plants in defending against potential herbivores [80,81]. According to the Optimal Defense Theory, plants, particularly those with extrafloral nectaries, secrete more nectar during periods of high herbivore activity and on their most vulnerable organs (e.g., young leaves) [46,82]. Most studied plants secrete nectar at the peak of flowering to reward pollinators and improve pollination [83]. *Tococa guianensis* possesses extrafloral nectaries that secrete nectar at the tips of the petals while the bud is still developing. Nectar production halts after flowering. Research indicates that nectar secretion from buds attracts ants, which helps reduce herbivorous insect feeding on flowers [5]. *Hemerocallis citrina* possesses extrafloral nectaries on its sepals and secretes nectar prior to flowering to attract predatory insects for defense [6]. In this study, *P. lactiflora* has been shown to secrete abundant nectar during the early bud stage, attracting ants to the bracts and sepals surrounding the buds for protection. Nectar secretion subsequently halts during the flowering period. This may also indicate a protective mechanism. Occasional visits by bees were observed during the secretion of its nectar. However, since nectar secretion occurs before bud opening, these visits do not serve a pollination purpose and thus constitute nectar-stealing behavior.

Research indicates that calcium oxalate crystals are frequently found in close proximity to, or within, nectary tissues, thereby enhancing their mechanical resistance to herbivore damage [84]. The presence of these crystals also offers structural support to nectary tissues, thereby improving the capacity of the phloem to transport substances [84]. Trichomes act as a physical barrier for plants, and their presence in EFNs prevents damage from small herbivores [85]. Calcium oxalate crystals were frequently observed in the non-nectar-producing regions of the bracts of *P. lactiflora* cultivars ‘Lan Hai Bi Bo’, ‘Jin Xing Shan Shuo’, and ‘Qi Hua Lu Shuang’ during stages S2–S5. Calcium oxalate crystals were occasionally noted in the tissues of the cultivar ‘Yang Fei Chu Yu’. The epidermal trichomes were non-glandular and lacked secretory functions, similar to the trichomes on the nectary epidermis of *Miconia tococa*; both perform defensive roles [86]. Defensive pathways may differ among various *P. lactiflora* cultivars.

Research indicates that, as a key signaling molecule, methyl jasmonate (MeJA) enhances sugar transport and accumulation in nectar glands during the bud stage of *P. lactiflora* [87]. This is accomplished by activating the expression of sugar transporter genes, improving the activity of enzymes involved in sucrose synthesis, and optimizing the allocation of carbon sources. Additionally, it regulates the flow of carbohydrates and secondary metabolites to shape nectar composition and expedite secretion. Moreover, MeJA activates plant defense responses by inducing the synthesis of defensive compounds, including protease inhibitors and antimicrobial proteins, in nectar. It also attracts natural enemies, such as ants, through the nectar, thereby establishing an interactive defense system [39,88]. In this study, the application of MeJA promoted nectar secretion, which indirectly suggests that nectar secretion during the bud stage may serve a defensive function.

## 5. Conclusions

The nectar-secreting sites of *P. lactiflora* are located along the abaxial margins at the base of the floral bud bracts and along the abaxial margins of the sepals. These sites constitute structural nectaries and are of the non-starch-storing type. The quantity of nectar secreted varies among different cultivars, and significant positive correlations exist between nectary stomatal aperture, the percentage of nectar-secreting stomata, and nectar secretion. The close association between the stomata and the underlying vascular tissue establishes the structural basis for its nectar secretion, whereas the photosynthetic potential of the nectaries themselves offers localized energy support for sustained nectar production during the bud stage. These findings enhance fundamental knowledge regarding *P. lactiflora* nectar secretion and establish a theoretical foundation for the future development of efficient regulatory technologies.

## Figures and Tables

**Table 1:** Basic information of 32 *P. lactiflora* cultivars.

Serial Number	Cultivar	Flowering Period	Flower Color	Flower Type
1	‘Dong Fang Shao Nv’	L	I	d
2	‘Yang Fei Chu Yu’	L	I	b
3	‘Zi Feng Yu’	E	III	e
4	‘Lan Tian Piao Xiang’	M	II	d
5	‘Lan Hai Bi Bo’	L	II	d
6	‘Xiang Tian Ge’	E	II	c
7	‘Jin Dai Huan Jiao’	M	II	d
8	‘Feng Huang Nie Pan’	E	I	a
9	‘Fen Chi Jin Yu’	M	I	d
10	‘Hong Xiu Qiu’	L	III	c
11	‘Qi Hua Lu Shuang’	E	II	e
12	‘Jin Xing Shan Shuo’	M	III	e
13	‘Yan Zi Xiang Yang’	E	III	d
14	‘Da Fu Gui’	E	III	d
15	‘Xue Feng’	M	I	c
16	‘Gao Gan Hong’	E	III	d
17	‘Kun Shan Xia Guang’	M	I	b
18	‘Hong Cha Hua’	M	III	d
19	‘Zhao Yuan Hong’	M	III	c
20	‘Xue Shan Zi Yu’	L	I	d
21	‘Lan Ju’	M	II	e
22	‘Hong Hua Lu Shuang’	M	III	b
23	‘Chi Fen’	L	II	b
24	‘Da Fang Yi Cai’	L	II	a
25	‘Die Lian Hua’	M	III	e
26	‘Fen Lan Ju Yun’	M	II	b
27	‘Huan Yan’	M	III	b
28	‘Hui Cui’	M	III	c
29	‘Ling Bo Zui’	E	II	a
30	‘Mei Ju’	E	II	e
31	‘Ran Fang Sai’	E	I	a
32	‘Yang Wang Xing Kong’	L	III	c

Note: The flowering period was divided into early-, mid-, and late-flowering [23], represented as E, M, and L, respectively; flower color was divided into light, medium, and dark [24,25], represented as I, II, and III, respectively; flower type [23] was divided into a. simple form; b. rose or chrysanthemum form; c. crown or globular form; d. prolification form; e. anemone form.

**Table 2:** Spearman’s correlation coefficients between nectar secretion index and flowering period/flower color/flower type.

Phenotypic Indicators	Correlation Coefficient/|*r*|		
	Flower Period	Flower Color	Flower Type
Nectar secretion index	0.088	0.075	0.116

**Table 3:** Detection of sugar content of *P. lactiflora* nectar based on GC-MS.

Number	Index	Class	Sugar Content mg/g	
			LHBB (High Production)	YFCY (Low Production)
1	1,5-Anhydroglucitol	Monosaccharide	-	-
2	2-Acetamido-2-deoxy-Dglucopyranose	Monosaccharide	0.048 ± 0.009	0.068 ± 0.001
3	2-Deoxy-D-ribose	Monosaccharide	-	-
4	Barium D-ribose-5-phosphate	Monosaccharide	0.077 ± 0.011	0.094 ± 0.013
5	Cellobiose	Disaccharide	0.098 ± 0.017	0.106 ± 0.016
6	D-Arabinitol	Monosaccharide	0.009 ± 0.001	0.014 ± 0
7	D-Arabinose	Monosaccharide	-	-
8	Deoxyglucose	Monosaccharide	-	-
9	D-Fructose	Monosaccharide	0.798 ± 0.02	0.823 ± 0.022
10	D-Galactose	Monosaccharide	0.01 ± 0	0.013 ± 0.001
11	D-Galacturonic acid	Monosaccharide	0.009 ± 0.001	0.009 ± 0
12	D-Glucuronic acid	Monosaccharide	-	-
13	D-Mannose	Monosaccharide	0.01 ± 0	0.009 ± 0
14	D-Mannose-6-phosphate sodium salt	Monosaccharide	0.392 ± 0.017	0.264 ± 0.038
15	D-Ribono-1,4-lactone	Monosaccharide	-	-
16	D-Ribose	Monosaccharide	0.004 ± 0.001	0.01 ± 0.005 *
17	D-Sorbitol	Monosaccharide	0.01 ± 0.002	0.02 ± 0.001
18	D-Xylose	Monosaccharide	0.043 ± 0.009	0.065 ± 0.018
19	D-Xylulose	Monosaccharide	0.003 ± 0.001	0.011 ± 0.001 *
20	Glucose	Monosaccharide	0.617 ± 0.018	0.671 ± 0.014
21	Inositol	Monosaccharide	15.681 ± 1.772	16.198 ± 1.444
22	Lactose	Disaccharide	-	-
23	Levoglucosan	Monosaccharide	1.066 ± 0.111	1.253 ± 0.086
24	L-Fucose	Monosaccharide	0.014 ± 0.002	0.015 ± 0.001
25	L-Rhamnose	Monosaccharide	0.01 ± 0.001	0.01 ± 0
26	Maltose	Disaccharide	1.116 ± 0.169	1.098 ± 0.129
27	Methylgalactoside	Monosaccharide	-	-
28	Phenylglucoside	Disaccharide	0.006 ± 0	0.007 ± 0
29	Raffinose	Trisaccharide	2.431 ± 0.118	2.659 ± 0.131
30	Sucrose	Disaccharide	307.98 ± 4.449	357.44 ± 8.022
31	Trehalose	Disaccharide	0.034 ± 0.005	0.05 ± 0.005
32	Xylitol	Monosaccharide	-	-

Note: The data presented in the table are expressed as the mean ± standard deviation. The symbol ‘*’ denotes a significant difference for that item (fold change ≥ 2 or ≤0.5).

**Table 4:** The characteristics of the stomata in the nectary epidermis of four studied *P. lactiflora* cultivars at the S3 stage.

Tested Feature		Cultivars							
		‘Lan Hai Bi Bo’		‘Jin Xing Shan Shuo’		‘Yang Fei Chu Yu’		‘Qi Hua Lu Shuang’	
		Bract	Sepal	Bract	Sepal	Bract	Sepal	Bract	Sepal
SL	(μm)	36.26 ± 1.5 ^Aa^	33.02 ± 1.92 ^ABb^	33.59 ± 2.96 ^Ba^	33.79 ± 6.06 ^ABa^	34.09 ± 1.8 ^Ba^	34.37 ± 2.92 ^Aa^	37.41 ± 3.7 ^Aa^	31.55 ± 4.75 ^Bb^
SW	(μm)	29.59 ± 2.58 ^Aa^	26.71 ± 2.08 ^Ab^	27.25 ± 3.67 ^Ba^	25.63 ± 5.06 ^Aa^	25.51 ± 1.97 ^Cb^	27.75 ± 2.54 ^Aa^	21.45 ± 1.94 ^Da^	22.21 ± 3.31 ^Ba^
SA’	(μm^2^)	811.8 ± 41.28 ^Aa^	741.78 ± 62.04 ^Ab^	786.59 ± 139.7 ^ABa^	650.99 ± 183.94 ^Bb^	710.25 ± 34.98 ^Cb^	755.26 ± 71.67 ^Aa^	742.21 ± 94.17 ^BCa^	543.68 ± 111.27 ^Cb^
SA	(μm)	8.65 ± 0.84 ^Ba^	6.91 ± 0.71 ^Bb^	10.07 ± 1.44 ^Aa^	9.24 ± 2.25 ^Ab^	4.84 ± 0.27 ^Da^	4.74 ± 0.57 ^Da^	5.43 ± 0.94 ^Ca^	5.81 ± 1.13 ^Ca^
SD/mm^2^	(pc.)	87.18 ± 6.92 ^Ba^	73.33 ± 5.72 ^Bb^	54.84 ± 8.36 ^Db^	65.59 ± 6.3 ^Ca^	140.51 ± 9.85 ^Aa^	121.54 ± 7.8 ^Ab^	76.56 ± 7.56 ^Ca^	76.13 ± 3.82 ^Ba^
SS	(pc.)	64.33 ± 6.58 ^Ba^	54.96 ± 4.64 ^Bb^	70.3 ± 5.93 ^Ab^	74.37 ± 3.28 ^Aa^	29.22 ± 3.48 ^Ca^	29.54 ± 0.75 ^Ca^	18.65 ± 6.81 ^Da^	22.39 ± 2.59 ^Da^

SL: stomatal length; SW: stomatal width; SA’: stomatal area; SA: stomatal aperture; SD: stomatal density; SS: percentage of nectar-secreting stomata. Identical letters indicate no significant difference (*p* > 0.05). The uppercase letters are the differences between different cultivars in the same part, and the lowercase letters are the differences between different parts of the same cultivar. The data shown in the figure are mean ± standard deviation.

**Table 5:** Spearman’s correlation coefficients between nectar secretion index and surface stomatal index of nectar secretion sites of different *P. lactiflora* cultivars.

		Correlation Coefficient					
Nectary	Phenotypic	SL	SW	SA’	SA	SD	SS
Bract	Nectar secretion index	0.046	0.711 **	0.427	0.711 **	−0.148	0.725 **
Sepal		−0.105	0.084	0.238	0.666 *	−0.148	0.725 **

‘*’ represents a significant correlation at the 0.05 level, and ‘**’ represents a highly significant correlation at the 0.01 level.

## Data Availability

The datasets generated or analyzed during the current study are available from the corresponding author on request.

## Supplementary Materials

The following supporting information can be downloaded at: https://www.mdpi.com/article/10.3390/plants15040580/s1, Figure S1: Schematic diagram illustrating the measurement of stomatal parameters. Note, SL: stomatal length; SW: stomatal width; SA’: stomatal area; SA: stomatal aperture.
