# Supplementary material for: Nectary Structure and Nectar Secretion Characteristics Among Various Cultivars of Paeonia lactiflora
Source: Plants (Basel). 2026 Feb 12;15(4):580. doi: 10.3390/plants15040580 (PMC12944572; doi:10.3390/plants15040580)
Supplement: Supplementary file 1 [file plants-15-00580-s001.zip › plants-4109268-supplementary.pdf]

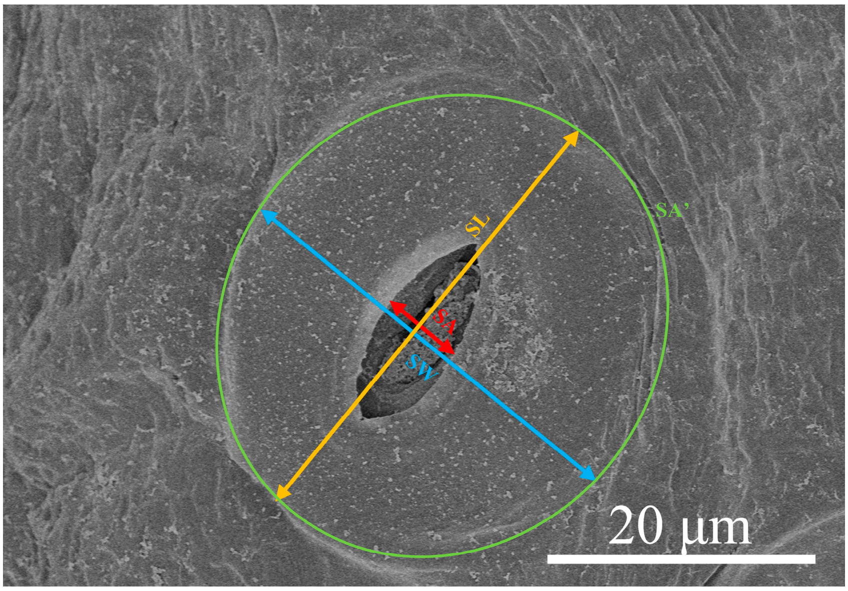

Figure S1. Schematic diagram illustrating the measurement of stomatal parameters. Note, SL: stomatal length; SW: stomatal width; SA': stomatal area; SA: stomatal aperture.
